# The Relation of Pain to Swallowing

**Published:** 1893-11

**Authors:** A. McNeil

**Affiliations:** San Francisco, Cal.


### THE RELATION OF PAIN TO SWALLOWING.

A. McNEIL, M. D., SAN FRANCISCO, CAL.

Swallowing relieves the pain, Alum., Caps., Ignat., Lach., Mang., Nux-v., Puls.

The distinction is finer in tracing out the different kinds of swallowing. For example:

Painful swallowing when it is empty or merely swallowing of saliva indicates Bar-c., Bell., Bry., Cocc., Hep., Lach., Merc., Nux-v., Puls., Rhus, Sulph.

On the other hand painful swallowing of food: Alum., Am-mur., Apis, Bar-c., Brom., Bry., Cham., Cocc., Coff., Hep., Lach., Merc., Nitr-ac., Nux-v., Phosph., Rhus, Sulph.

Among the latter the painful swallowing of solid food especially indicates Cham., Hep., Lach., Merc., Sulph., and that from liquid food and drinks, Bell., Brom., Ignat., Merc., Phosph. But when, on the other hand, the swallowing of liquids ameliorates it points to Alum., Nitr-ac., Nux-v., and when this amelioration is particularly from warm liquids, Alum. and Nux-v. lead all other remedies. Nearly related to swallowing is talking. When this aggravates the pains in the throat it indicates more particularly Acon., Alum., Bar-c., Bry., Dulc., Ignat., Mang., Merc., Phosph., Rhus, Sulph. And when this rare case occurs when it (speaking) improves it points almost exclusively to Hepar.

